# Dietary* Saccharomyces cerevisiae* Cell Wall Extract Supplementation Alleviates Oxidative Stress and Modulates Serum Amino Acids Profiles in Weaned Piglets

**DOI:** 10.1155/2017/3967439

**Published:** 2017-03-12

**Authors:** Gang Liu, Lei Yu, Yordan Martínez, Wenkai Ren, Hengjia Ni, Naif Abdullah Al-Dhabi, Veeramuthu Duraipandiyan, Yulong Yin

**Affiliations:** ^1^Key Laboratory of Agro-Ecological Processes in Subtropical Region, Institute of Subtropical Agriculture, Chinese Academy of Sciences, National Engineering Laboratory for Pollution Control and Waste Utilization in Livestock and Poultry Production, Hunan Provincial Engineering Research Center of Healthy Livestock, Scientific Observing and Experimental Station of Animal Nutrition and Feed Science in South-Central, Ministry of Agriculture, Hunan Co-Innovation Center of Animal Production Safety, Hunan 410125, China; ^2^China Animal Disease Control Center, Tiangui Street No. 17, Daxing District, Beijing 102600, China; ^3^Study Center of Animal Production, Faculty of Veterinary Medicine, University of Granma, Bayamo, 85100 Granma, Cuba; ^4^Addiriyah Chair for Environmental Studies, Department of Botany and Microbiology, College of Science, King Saud University, P.O. Box 2455, Riyadh 11451, Saudi Arabia; ^5^Laboratory of Animal Nutrition and Human Health, School of Biology, Hunan Normal University, Changsha, Hunan, China; ^6^College of Animal Science, South China Agricultural University, Guangzhou 510642, China

## Abstract

This research aims to evaluate the effects of dietary supplementation with* Saccharomyces cerevisiae* cell wall extract (SCCWE) on growth performance, oxidative stress, intestinal morphology, and serum amino acid concentration in weaned piglets. Utilizing a completely randomized design, 40 healthy piglets weaned at 21 d were grouped into 4 experimental treatments with 10 pigs per treatment group. Treatments consisted of a basal diet (T0), a basal diet with a 0.05% SCCWE (T1), a basal diet with a 0.10% SCCWE (T2), and a basal diet with a 0.15% SCCWE (T3). SCCWE supplementation increased the average daily gain and final body weight compared with T0 (*P* < 0.05). SCCWE in T2 and T3 improved the average daily feed intake and decreased the feed/gain ratio compared with T1 and T2 (*P* < 0.05). SCCWE decreased serum malondialdehyde (MDA) and increased activities of catalase (CAT), glutathione peroxidase (GPx), and superoxide dismutase (SOD) significantly compared to T0 (*P* < 0.05). SCCWE increased the concentration of Ile compared to T0 (*P* < 0.05). Moreover, the concentrations of Leu, Phe, and Arg were higher in T2 and T3 (*P* < 0.05). These findings indicate beneficial effects of SCCWE supplementation on growth performance, the concentration of some essential amino acids, and alleviation of oxidative stress in weaned piglets.

## 1. Introduction

Weaning is the most important stress in the pig farming industry, and it deeply affects gut health and the immune system. During the first weeks after weaning, piglets need to adjust to solid feed [1]. As a consequence, weaning may result in decreased feed intake, and piglets may undergo serious diarrhea and immune dysfunction. In weaned piglets, environmental factors can also result in oxidative stress. When the environment changes, the balance between oxidation and antioxidation is disturbed, resulting in mass production of reactive oxygen species (ROS) in the body, eventually leading to oxidative stress. ROS such as superoxide and H_2_O_2_ [2, 3] are constantly produced from oxygen during the body's metabolic processes. Under normal conditions, ROS are maintained at certain levels, and excessive oxidative radicals are usually removed by a series of antioxidant enzymes such as glutathione peroxidase (GPx), catalase (CAT), and superoxide dismutase (SOD).

Yeast cell wall is rich in prebiotics and has been studied in pig production, mainly focusing on its effects on growth performance, immune modulation, and microbiology [4–6]. As a result, several reports have suggested that it is capable of promoting growth performance. However, the animal response results are relatively variable [4]. Li and Kim have demonstrated that supplementation with 0.10% SCCWE improved the growth performance and digestibility, modulated the fecal microbiota, and decreased the emissions of fecal gas in growing pigs [7]. However, Sauerwein et al. found that feeding pigs with SCCWE did not increase growth performance and that the immunomodulatory response was mild [4].

However, to our knowledge, only a few investigations have been conducted involving yeast extract on piglet diets, with the purpose of finding ways to reconcile the scientific contradictions on the topic. We hypothesized that dietary supplementation with* Saccharomyces cerevisiae* cell wall extract could improve growth performance by decreasing oxidative stress and increasing the circulation of nutrients and intestinal health in piglets. Thus, the objective of this study was to evaluate the effect of dietary* Saccharomyces cerevisiae* cell wall extract supplementation on growth performance, oxidative stress, intestinal morphology, and serum amino acid concentration in weaned piglets.

## 2. Materials and Methods

### 2.1. Animal, Housing, and Treatment

The experiment conformed to the guidelines for animal welfare and experimental protocol of the Institute of Subtropical Agriculture, Chinese Academy of Sciences. A total of 40 healthy piglets [(Yorkshire × Landrace) × Duroc] weaned at 21 d were obtained from a local commercial swine herd. Piglets were randomly grouped into four groups based on their body weight (BW) and placed into 40 pens (length, 0.5 m; width, 0.4 m; height, 0.5 m) with 10 replicates per group [8].

After a five-day adjustment, piglets were separately fed with one of four kinds of diets (Table 1): basal diet (T0), basal diet + 0.05%* Saccharomyces cerevisiae* cell wall extract (T1), basal diet + 0.10%* Saccharomyces cerevisiae* cell wall extract (T2), and basal diet + 0.15%* Saccharomyces cerevisiae* cell wall extract (T3). The* Saccharomyces cerevisiae *cell wall extract (Antaferm-MG; Dr. Eckel GmbH, Niederzissen, Germany) was prepared according to a previous study [4]. These diets were formulated to meet or exceed the NRC (2012) nutrient specifications for pigs weighing 5 to 20 kg and had similar ingredients and nutrient levels. All piglets were given free access to feed and water for 21 d.

### 2.2. Growth Performance and Diarrhea Rate

The initial and final body weights and feed consumption were recorded during the whole trial. Based on these data, the average daily gain (ADG), average daily feed intake (ADFI), and feed/gain (F : G) ratio were calculated according to the method of Liu et al. [9].

The diarrhea rate for the piglets was calculated as follows:

(1) $$\begin{array}{c} Diarrhea\;\;rate=\left[\;\frac{\left(number\;\;of\;\;piglets\;\;with\;\;diarrhea\times number\;\;of\;\;days\;\;of\;\;diarrhea\right)}{\left(total\;\;number\;\;of\;\;piglets\times number\;\;of\;\;days\;\;of\;\;experiment\right)}\;\right]\times 100\%. \end{array}$$

### 2.3. Serum Antioxidative Enzymes and Amino Acids

On day 21 of the trial, 10 mL of blood was drawn from the jugular vein into plastic uncoated tubes. After collection, blood samples were centrifuged at 8,000 rpm and 4°C for 10 min. Serum was then collected and frozen at −20°C for further analysis. MDA, CAT, GPx, and SOD were analyzed with kits purchased from Nanjing Jiancheng Bioengineering Institute according to the manufacturer's protocol. A 7.5% trichloroacetic acid solution (2.5 mL) was added to 1 mL serum and mixed thoroughly. This mixture was centrifuged at 12000 rpm and 4°C for 15 min. The supernatant was collected and applied to an ion-exchange AA analyzer (Hitachi L-8900 Auto-Analyzer, Tokyo, Japan) for the determination of amino acids.

### 2.4. Sampling and Morphological Analyses

At the end of the experiment, all piglets were killed and small intestine samples were collected. For histomorphological analysis, the duodenum, jejunum, and ileum tissues (5 cm) were fixed with 10% formalin in PBS at 4°C, dehydrated in a graded series of ethanol, and then embedded in paraffin wax. Then, they were sectioned at 5 *μ*m thickness and mounted on slides; this was followed by dewaxing, hydrating, and staining the tissues with Hematoxylin-Eosin. The villus height (VH) and crypt depth (CD) were measured using an Axiostar plus microscope (Carl Zeiss, Oberkochen, Germany). The VH/CD ratio (VCR) was then calculated.

### 2.5. Statistical Analyses

The results were analyzed by one-way ANOVA using SPSS 16.0 software (SPSS Inc., Chicago, IL, USA). Duncan's multiple range test was used to compare differences among different groups. *P* < 0.05 was considered statistically significant.

## 3. Results

Data on the effect of dietary supplementation with* Saccharomyces cerevisiae* cell wall extract (SCCWE) on growth performance and diarrhea rate are presented in Table 2. SCCWE supplementation increased the final BW and ADG and reduced the diarrhea rate compared with T0 (*P* < 0.05). Similarly, T2 and T3 improved the ADFI (*P* < 0.05); meanwhile, T1 did not differ significantly with T0 (*P* > 0.05). The feed/gain ratio decreased in T1 and T2 compared to T0 (*P* < 0.05). However, the feed/gain ratio of T0 and T3 were not significantly different (*P* > 0.05).

Data on MDA, CAT, SOD, and GPx are shown in Table 3. Compared with the control group, SCCWE supplementation decreased MDA in the serum (*P* < 0.05), while SCCWE supplementation in T1, T2, and T3 increased CAT, SOD, and GPx (*P* < 0.05). However, there were no significant differences among T1, T2, and T3 (*P* > 0.05).

On day 21 (Table 4), the concentrations of Ile, Leu, Phe, and Arg were changed by the effect of experimental treatments with SCCWE. The other amino acids did not show significant differences between groups (*P* > 0.05). SCCWE supplementation up to 0.15% increased the concentration of Ile compared to T0 (*P* < 0.05). Moreover, the levels of Leu and Phe were higher in T2 and T3 compared to T0 and T1 (*P* < 0.05). Additionally, T2 and T3 increased the concentration of Arg compared to T0 (*P* < 0.05).

Table 5 shows the effect of* Saccharomyces cerevisiae* cell wall extract supplementation on small intestinal morphology in weaned piglets. The villus height of the jejunum and ileum increased gradually (*P* < 0.05) with SCCWE supplementation. However, the duodenum villus height did not show significant differences (*P* > 0.05) among treatments. Similarly, SCCWE supplementation did not change (*P* > 0.05) the crypt depth of the previously mentioned intestinal regions (Table 5). Nevertheless, the villus height : crypt depth ratio in jejunum and ileum increased significantly in T3 (*P* > 0.05) compared with T0.

## 4. Discussion 

*Saccharomyces cerevisiae* yeast is a rich source of prebiotics, mainly *β*-glucan. Several studies have reported beneficial effects of *β*-glucans on growth performance and health in pigs. Kogan and Kocher found that SCCWE polysaccharides (mainly *β*-glucan) can modulate immunity and the absorption of mycotoxin, prevent the adhesion and dissemination of bacteria, and finally improve the health of pigs [10]. Additionally, Sweeney et al. showed that oral administration of *β*-glucans extracted from* Saccharomyces cerevisiae* increased the growth performance of piglets [11]. Additionally, Li and Kim reported an increase in BW and feed intake in growing pigs fed with SCCWE, attributed to its beneficial effect on lactic acid bacteria [7]. The present results showing that* Saccharomyces cerevisiae* cell wall extract supplementation up to 0.15% led to the highest BW suggest that this product may enhance the growth performance of pigs, a finding corroborated by Li and Kim [7], who attributed these responses to its beneficial effects on immunity, microbiology, feed intake, and digestion. Thus, our results find a growth promoting effect of* Saccharomyces cerevisiae* cell wall extract in piglets.

Another novel and important point in this study is that SCCWE supplementation reduces the diarrhea rate in weaned piglets (Table 2). Diarrheal syndrome, which causes the loss of water and electrolytes in semiliquid and liquid feces, occurs mainly due to the proliferation of* Escherichia coli* and other pathogenic bacteria in the gut after weaning [12]. There is a relationship between fecal pH, the intestinal microflora population, and the diarrhea rate of pigs [13]. Stabilization of intestinal pH at a reasonably low level and maintaining an optimal balance of intestinal microbiota both play major roles in decreasing the diarrhea rate [14].

*β*-Glucans are capable of activating the innate immune system, thereby reinforcing defense barriers and preventing pathogen infection. Some studies report that the polysaccharides in yeast cell wall have been used against various bacterial infections and yeast, fungal, viral, and parasitic diseases. In this sense, Stuyven et al. testified that *β*-glucans from* Saccharomyces cerevisiae* as a feed ingredient could protect against an ETEC infection [15]. Moreover, Li and Kim found a decrease of* Escherichia coli* and an increase of* Lactobacillus* when they used SCCWE and poplar propolis ethanol extract in pig diets [7]. Thus, considering our result that SCCWE supplementation greater than 0.05% decreased the diarrhea rate (Table 2), we can affirm an antidiarrheal effect of yeast cell wall extract in weaned piglets. However, studies in which (1-3)(1-6)-*β*-glucans are tested to protect piglets against diarrheal syndrome in pigs are few.

Many factors could contribute to cell damage due to oxidative stress in pig production. Oxidative stress may lead to worsening health status and decreased growth performance, as Yuan et al. described that growth performance was impaired in piglets under oxidative stress [16]. In the present study, the concentration of MDA in plasma was also higher in T0 than T1, T2, and T3. MDA is a metabolite produced after lipid peroxidation, and with the thiobarbituric acid assay, we can analyze lipid peroxidation and products. Cat, SOD, and GPx are the main antioxidant enzymes that can be used to monitor antioxidative capability in piglets [17]. In this research, the activities of these enzymes in serum were significantly increased in the SCCWE groups, suggesting that the antioxidative capabilities of piglets were increased. Moreover, we found that dietary supplementation of SCCWE significantly decreased the concentration of MDA, indicating that supplementation of SCCWE can effectively relieve the oxidative stress of piglets.

According to Wu et al., an increase in the amount of amino acids that enter the portal vein can be useful in promoting tissue protein synthesis in animals [18]. Similar to other investigations with nutraceutical foods on pig diets [13], we found a higher serum amino acid concentration, mainly essential amino acids. In this sense, the leucine that increased with SCCWE supplementation together with isoleucine and valine intervened in the formation and repair of muscle tissue, helping to protect muscles and regulating blood sugar levels. It also acts as a good energy source and helps increase production of growth hormone, which is important in the intensive production of pigs [18]. Additionally, isoleucine, which increased at concentrations of SCCWE greater than 0.05% (Table 4), participates in the formation of hemoglobin and regulates and stabilizes blood sugar and energy levels. Furthermore, isoleucine helps in repairing muscle tissue, skin, and bones [8].

Generally, the use of prebiotics, probiotics, and herbs in pig diets increases the concentration of arginine, leading to a better animal response [19], as is evident in our results (Table 4). Apparently, improved intestinal health could help to improve the content of this amino acid. Arginine is a vital amino acid in metabolism and participates in multiple pathways, including the synthesis of proteins, polyamines, NO, and creatine. Moreover, Tan et al. (2009) demonstrated that arginine reduces body fat mass and increases muscle gain by its effects on endothelial vasodilators. Similarly, this amino acid plays a role in T-lymphocyte proliferation in response to mitogens [20].

Another amino acid that increased (*P* < 0.05) with SCCWE supplementation on day 21 after initiation of the experiment was phenylalanine, which is an important precursor for the synthesis of acetoacetyl-CoA and tyrosine. In the intestine, phenylalanine acts as a competitive inhibitor in the regulation of amino acid absorption. Some interactions between phenylalanine and the anionic amino acids, aspartate and glutamate, are also observed during metabolic processes [21]. Phenylalanine oxidation is used to indicate the partition of absorbed amino acids between protein synthesis and oxidation.

Intestinal morphological structure is affected by diet and intestinal health. The intestinal villus and crypt are correlated with gut health and growth in pigs [22]. Therefore, our results suggest a beneficial effect of SCCWE supplementation (from 0.10%) on growth performance and on the villus height of the jejunum and ileum. Similarly, Zhang et al. have reported a higher villus height in birds fed with yeast cell wall compared to a control treatment, but there was no significant change in crypt depth in the small intestine [23]. Apparently this product (SCCWE) did not affect the crypt depth, possibly because fiber contributions (Table 1) did not surpass the permissible limits for the category and animal species, and high fiber diets cause a reduction of feed intake with a lower crypt depth in pigs [24]. In pigs, there have been few studies noting the benefits of yeast cell walls on intestinal morphology.

Although the composition or the activity of the microflora in the digestive tract was not determined in this study, other studies have indicated that SCCWE supplementation reduced the population of* E. coli* [7] and increased lactic acid bacteria. Thus, the weight of the small intestine might be reduced by decreasing inflammation with better development of intestinal villi, in our case, in the jejunum and ileum (Table 5). However, other authors using dietary additives have found an increased villus height of the duodenum, despite the fact that it generally atrophies more seriously than the jejunum and ileum after weaning [25].

These findings show a beneficial dietary effect of* Saccharomyces cerevisiae* cell wall extract supplementation on growth performance, the serum concentration of some essential and nonessential amino acids, and intestinal morphology. Additionally, these results show that this product is safe and effective in reducing diarrhea, and it may be used as a substitute for antibiotics in weaned piglets.

## Figures and Tables

**Table 1:** Dietary ingredients and nutrient levels in the diets (as fed).

Ingredients (g kg^−1^)	T0	T1	T2	T3
Corn	530.0	529.5	529.0	528.5
*Saccharomyces cerevisiae* cell wall extract	—	0.5	1.0	1.5
Soybean meal	220.0	220.0	220.0	220.0
Wheat bean	50.0	50.0	50.0	50.0
Fish meal	50.0	50.0	50.0	50.0
Soybean oil	30.0	30.0	30.0	30.0
Dried porcine soluble	25.0	25.0	25.0	25.0
Sucrose	15.0	15.0	15.0	15.0
Glucose	15.0	15.0	15.0	15.0
Lysine	4.0	4.0	4.0	4.0
Threonine	8.0	8.0	8.0	8.0
Salt	4.0	4.0	4.0	4.0
Limestone	7.0	7.0	7.0	7.0
Calcium hydrophosphate	22.0	22.0	22.0	22.0
Premix^†^	20.0	20.0	20.0	20.0
Calculated composition				
Digestive energy (MJ kg^−1^)	14.8	14.8	14.8	14.8
Crude protein (g kg^−1^)	215.0	215.0	215.0	215.0
Calcium (g kg^−1^)	9.2	9.2	9.2	9.2
Phosphorus (g kg^−1^)	6.7	6.7	6.7	6.7

^†^Supplied per kg diet: 10 mg copper; 100 mg iron; 0.3 mg sodium; 100 mg zinc; 10 mg manganese; 386 IU cholecalciferol; 3086 IU retinyl acetate; 15.4 IU all-rac-*α*-tocopheryl acetate; 2.3 mg menadione; 3.9 mg riboflavin; 15.4 mg D-pantothenic acid; 23 mg niacin; 77 mg choline; and 15.4 *μ*g cyanocobalamin.

**Table 2:** Effect of dietary supplementation with extract of *Saccharomyces cerevisiae* cell wall on growth performance and diarrhea rate in weaned piglets.

Items	T0	T1	T2	T3	SEM±	*P* value
Initial BW (kg)	5.76	5.76	5.75	5.75	0.43	0.88
Final BW (kg)	12.75^b^	13.34^a^	13.65^a^	13.60^a^	0.56	0.042
ADG (g)	333.0^b^	361.0^a^	376.0^a^	374.0^a^	49.0	0.035
ADFI (g d^−1^)	536.0^b^	549.0^ab^	568.0^a^	583.0^a^	63.0	0.018
F/G	1.61^b^	1.52^a^	1.51^a^	1.56^ab^	0.11	0.036
Diarrhea rate (%)	21.60	18.75	17.45	18.24		

^a,b^Means in the same row with different superscripts are significant (*P* < 0.05).

**Table 3:** Effect of dietary supplementation with extract of *Saccharomyces cerevisiae* cell wall on MDA and antioxidative enzymes.

Items	T0	T1	T2	T3	SEM±	*P* value
MDA (nmol/mL)	1.83^a^	1.45^b^	1.51^b^	1.48^b^	0.12	0.035
CAT (U/mL)	125.8^a^	143.2^b^	152.1^b^	150.4^b^	8.5	0.030
SOD (U/mL)	68.50^a^	75.1^b^	77.4.0^b^	77.8^b^	5.4	0.038
GPx (U/mL)	630.8^a^	734.3^b^	728.4^b^	719.2^b^	43.6	0.011

^a,b^Means in the same row with different superscripts are significant (*P* < 0.05).

**Table 4:** Effect of dietary supplementation with extract of *Saccharomyces cerevisiae* cell wall on serum amino acids concentration (*μ*g/mL) in weaned piglets.

Items	T0	T1	T2	T3	SEM±	*P* value
Asp	19.07	18.63	19.12	18.96	1.32	0.457
Thr	71.90	70.63	72.94	71.55	3.52	0.675
Ser	40.01	42.32	41.48	40.96	1.86	0.447
Glu	102.77	103.56	103.27	102.96	8.12	0.463
Gly	84.30	78.85	83.28	85.96	7.54	0.521
Ala	143.24	155.32	147.25	148.62	7.63	0.216
Val	37.26	33.65	38.62	35.48	7.49	0.345
Met	91.47	88.74	92.13	86.61	8.65	0.425
Ile	24.63^b^	30.61^a^	31.57^a^	32.11^a^	2.56	0.033
Leu	49.88^c^	56.86^b^	66.81^a^	68.32^a^	2.31	0.014
Tyr	37.74	35.26	35.22	34.67	2.69	0.532
Phe	29.41^c^	32.41^b^	36.65^a^	37.21^a^	1.85	0.023
Lys	40.20	42.63	41.56	42.44	3.29	0.336
His	13.57	14.36	13.99	14.11	0.98	0.445
Arg	35.80^b^	39.52^ab^	43.67^a^	42.19^a^	3.11	0.043

^a,b,c^Means in the same row with different superscripts are significant (*P* < 0.05).

**Table 5:** Effect of dietary supplementation with extract of *Saccharomyces cerevisiae* cell wall on small intestinal morphology.

Items	T0	T1	T2	T3	SEM±	*P* value
Villus height, *μ*m						
Duodenum	398.6	403.6	406.8	406.5	9.12	0.153
Jejunum	399.1^c^	406.2^b^	413.4^a^	412.7^a^	10.58	0.024
Ileum	328.6^c^	348.2^b^	353.9^a^	352.1^a^	15.32	0.013
Crypt depth, *μ*m						
Duodenum	217.3	213.4	213.9	212.8	12.44	0.541
Jejunum	221.4	218.3	219.6	216.5	8.36	0.459
Ileum	208.1	206.5	205.7	206.4	7.65	0.612
Villus height: crypt depth						
Duodenum	1.83	1.89	1.90	1.91	0.12	0.217
Jejunum	1.80^b^	1.86^ab^	1.88^ab^	1.91^a^	0.08	0.035
Ileum	1.58^b^	1.69^ab^	1.72^ab^	1.71^a^	0.10	0.028

^a,b,c^Means in the same row with different superscripts are significant (*P* < 0.05).
